# Supplementary material for: Behavioural patterns of free roaming wild boar in a spatiotemporal context
Source: PeerJ. 2020 Nov 18;8:e10409. doi: 10.7717/peerj.10409 (PMC7680034; doi:10.7717/peerj.10409)
Supplement: Supplemental Information 1 [file peerj-08-10409-s001.docx]

**S1 Appendix**

**Significant results of the GAM (N > 20)**

**Locomotion** (N = 732)

| **Parameter** | **Estimate** | **p-value** | **edf** | **Deviance explained** |
| --- | --- | --- | --- | --- |
| non-track  track | -0,68 | < 0,001 *** | 7,97 | 10,10 % |
| forest aisle  non-forest aisle | 0,49 | 0,002 ** | 7,97 | 9,88 % |
| pond  non-pond | 1,62 | < 0,001 *** | 7,97 | 10,90 % |
| tree_broad-leaved forest  tree_coniferous forest  tree_mixed forest  tree_no trees | -0,38  -0,42  -0,70 | 0,005 **  0,022 *  > 0,05 | 7,97 | 9,86 % |
| shrub_broad-leaved forest  shrub_mixed forest  shrub_no shrubs  shrub_coniferous forest | -0,54  -0,60  -0,65 | 0,005 **  < 0,001 ***  < 0,001 *** | 7,97 | 10,00 % |
| herb_herbs  herb_P.a.&V.m.  herb_V.m.  herb_P.a.  herb_no herbs | 0,36  0,12  0,06  -0,23 | 0,047 *  > 0,05  > 0,05  > 0,05 | 7,97 | 10,10 % |
| herb_50-100 %  herb_0-50 %  herb_0 % | 0,58  0,24 | < 0,001 ***  > 0,05 | 7,97 | 10,10 % |
| deadwood_25-50 %  deadwood _0-25 %  deadwood _0 % | 0,41  0,02 | < 0,001 ***  > 0,05 | 7,97 | 10,10 % |
| no significant differences:  tree cover, shrub cover | | | | |

**Olfactory behaviour**

Sniffing and winding (N = 306)

| **Parameter** | **Estimate** | **p-value** | **edf** | **Deviance explained** |
| --- | --- | --- | --- | --- |
| non-track  track | -0,79 | 0,008 ** | 7,87 | 8,99 % |
| pond  non-pond | 2,71 | < 0,001 *** | 7,87 | 14,4 % |
| shrub_broad-leaved forest  shrub_no shrubs  shrub_mixed forest  shrub_coniferous forest | -0,21  -0,45  -0,72 | > 0,05  > 0,05  0,015 * | 7,87 | 8,94 % |
| herb_herbs  herb_AF  herb_HB  herb_no herbs  herb_AF&HB | 0,62  -0,09  -0,67  -0,91 | 0,019 *  > 0,05  0,033 *  > 0,05 . | 7,87 | 10,70 % |
| herb_50-100 %  herb_0-50 %  herb_0 % | 1,23  0,48 | < 0,001 ***  > 0,05 . | 7,87 | 10,30 % |
| deadwood_25-50 %  deadwood_0 %  deadwood_0-25 % | 0,53  -0,28 | 0,003 **  > 0,05 . | 7,87 | 9,44 % |
| no significant differences:  forest aisle, tree layer, tree cover, shrub cover | | | | |

**Vigilance behaviour** (N = 188)

| **Parameter** | **Estimate** | **p-value** | **edf** | **Deviance explained** |
| --- | --- | --- | --- | --- |
| forest aisle  non-forest aisle | 0,75 | < 0,001 *** | 7,87 | 8,44 % |
| pond  non-pond | 1,29 | < 0,001 *** | 7,87 | 8,45 % |
| tree_broad-leaved forest  tree_mixed forest  tree_coniferous forest  tree_no trees | -0,11  -0,41  -0,51 | > 0,05  0,047 *  > 0,05 | 7,87 | 8,12 % |
| shrub_broad-leaved forest  shrub_mixed forest  shrub_coniferous forest  shrub_no shrubs | -0,59  -0,75  -0,84 | 0,029 *  0,004 **  < 0,001 *** | 7,87 | 8,50 % |
| herb_50-100 %  herb_0-50 %  herb_0 % | 0,50  0,41 | 0,023 *  > 0,05 . | 7,87 | 8,19 % |
| deadwood_25-50 %  deadwood_0-25 %  deadwood_0 % | 0,47  0,13 | 0,002 **  > 0,05 | 7,87 | 8,40 % |
| no significant differences:  track, tree cover, shrub cover, herb layer | | | | |

**Foraging behaviour**

Rooting & pawing (N = 81)

| **Parameter** | **Estimate** | **p-value** | **edf** | **Deviance explained** |
| --- | --- | --- | --- | --- |
| forest aisle  non-forest aisle | 0,71 | 0,033 * | 7,89 | 8,64 % |
| pond  non-pond | 1,53 | < 0,001 *** | 7,89 | 9,03 % |
| tree_broad-leaved forest  tree_mixed forest  tree_coniferous forest  tree_no trees | -1,13  -1,36  -34,42 | 0,002 **  < 0,001 ***  > 0,05 | 7,89 | 10,90 % |
| shrub_broad-leaved forest  shrub_no shrubs  shrub_mixed forest  shrub_coniferous forest | -0,99  -1,05  -2,22 | 0,002 **  0,007 **  < 0,001 *** | 7,89 | 10,90 % |
| herb_herbs  herb_P.a.  herb_no herbs  herb_V.m.  herb_P.a.&V.m. | 0,81  -0,05  -0,47  -1,72 | 0,045 *  > 0,05  > 0,05  > 0,05 | 7,89 | 10,30 % |
| no significant differences:  track, tree cover, shrub cover, herb cover, deadwood | | | | |

Chewing & feeding (attempt) (N = 34)

| **Parameter** | **Estimate** | **p-value** | **edf** | **Deviance explained** |
| --- | --- | --- | --- | --- |
| pond  non-pond | 2,71 | < 0,001 *** | 7,80 | 12,3 % |
| shrub_50-100 %  shrub_0 %  shrub_0-50 % | 1,72  -1,40 | < 0,001 ***  0,015 * | 7,80 | 14,5 % |
| herb_V.m.  herb_herbs  herb_no herbs  herb_P.a.  herb_P.a.&V.m. | 1,99  0,96  0,31  -0,02 | 0,034 *  > 0,05  > 0,05  > 0,05 | 7,80 | 11,20 % |
| no significant differences:  track, forest aisle, tree layer, tree cover, shrub layer, herb cover, deadwood | | | | |

**Social interaction** (N = 26)

| **Parameter** | **Estimate** | **p-value** | **edf** | **Deviance explained** |
| --- | --- | --- | --- | --- |
| forest aisle  non-forest aisle | 1,38 | < 0,001 *** | 6,85 | 10,30 % |
| pond  non-pond | 1,41 | 0,042 * | 6,85 | 8,97 % |
| shrub_broad-leaved forest  shrub_mixed forest  shrub_coniferous forest  shrub_no shrubs | -0,69  -1,32  -2,10 | > 0,05  0,016 *  < 0,001 *** | 6,85 | 11,50 % |
| shrub_0-50 %  shrub_50-100 %  shrub_0 % | 1,37  1,24 | 0,015 *  > 0,05 | 6,85 | 10,20 % |
| herb_herbs  herb_P.a.&V.m.  herb_V.m.  herb_P.a.  herb_no herbs | 1,31  0,27  0,14  -39,84 | 0,006 **  > 0,05  > 0,05  > 0,05 | 6,93 | 15,10 % |
| deadwood_25-50 %  deadwood_0-25 %  deadwood_0 % | 1,23  0,64 | 0,007 **  0,019 * | 6,85 | 11,20 % |
| no significant differences:  track, tree layer, tree cover, herb cover | | | | |
